# Rapid and Simple Detection of* Trichosporon asahii* by Optimized Colony PCR

**DOI:** 10.1155/2019/1803278

**Published:** 2019-05-14

**Authors:** Dequan Zhang, Xuelian Lu, Yong Liao, Zhikuan Xia, Zhuoying Peng, Xin Yang, Rongya Yang

**Affiliations:** ^1^Army Medical University (Third Military Medical University), Chongqing 400038, China; ^2^The Military Institute of Injury and Reparation, The Seventh Medical Center of PLA General Hospital, Beijing 100700, China; ^3^Department of Dermatology, Beijing Anzhen Hospital, Capital Medical University, Beijing 100021, China

## Abstract

*Trichosporon asahii* is the major pathogen causing invasive trichosporonosis. Conventional methods of its detection are time-consuming or costly and often require complex DNA extraction and purification steps, which hinders rapid clinical diagnosis. In this study, we evaluated colony PCR, which directly uses colonies or trace clinical samples as the template for amplification, for rapid detection of* T. asahii* infection. Four methods, namely, direct colony, freeze-thaw, glass beads, and enzymolysis, were compared to select the best DNA extraction strategy. We subsequently designed and screened species-specific primers targeting the intergenic spacer 1 (IGS1) of the ribosomal DNA of* T. asahii* and used them to detect mock infection clinical samples. The species-specific colony PCR based on glass beads proved advantageous, with short procedure time (154.8 ± 0.6 min), good sensitivity (detection limit, 10^2^ CFU/mL), and specificity for* T. asahii*, indicating that this method can be used for the rapid and simple identification of clinical samples of* T. asahii* infection.

## 1. Introduction

Species of the* Trichosporon* genus exists widely in nature; the genus comprises 51 species, of which 16 are the pathogenic to humans. Following the genus* Candida*,* Trichosporon* is the most common pathogen among invasive yeast infections in patients with malignant hematological diseases [1].* T. asahii* is the most common pathogen among* Trichosporon* infections and can cause invasive fatal trichosporonosis, especially in immunocompromised hosts, such as individuals suffering from neutropenia, organ transplantation, hematologic malignancies, extensive burns, or AIDS[2].

The identification of* T. asahii* and diagnosis of infection with the pathogen often involves biochemical methods, conventional or real-time quantitative PCR, and mass-spectrometric methods. These are based on pathological biopsy specimens and fungal culture. Traditional biochemical methods are currently the most common approach for identification of* T. asahii* infection. However, this method depends on the isolation and purification of the isolate, and it is difficult to differentiate* T. asahii *strains from other closely related* Trichosporon* species [3, 4]. On the other hand, Matrix-Assisted Laser Desorption Ionization–Time of Flight Mass Spectrometry (MALDI-TOF MS) has already been applied for the identification of* T. asahii *[5]. However, only limited standard maps of the genus Trichosporon could be found in the original database, which made MALDI-TOF MS hard to be effectively and accurately applied for the identification of Trichosporon spp. Encouragingly, Kolecka and colleagues improved the accuracy and reliability of Trichosporon spp identification by extending reference maps of Trichosporon spp in MALDI-TOF database [6, 7]. Even so, this technology has limited application to pure cultures and the high cost of the equipment has limited its extensive clinical application to the hospital laboratories in developing countries.

The current fungal PCR amplification process is time-consuming and often requires traditional cell wall-breaking methods to extract DNA. It is difficult to obtain high quality DNA, especially when the fungal burden of the specimen is low, thus extremely limiting its application in clinical diagnosis. All of the above limitations have hindered the use of molecular identification and the application of PCR technology to clinical samples of* T. asahii* infection. While we have previously adapted loop-mediated isothermal amplification (LAMP) for identification of* T. asahii *[8], there is still no low-cost molecular method for* T. asahii* identification available for all levels of medical institutions worldwide, especially in developing countries [9].

In 1989, Gussow and Clackon first successfully applied the colony PCR method for the diagnosis of bacterial infection [10]. Because the bacterial cell wall is thin and weak, simple mechanical effects and high temperature are sufficient for their lysis and to release their DNA. There have also been reports on the application of colony PCR for the detection of fungi, but the quality of the resulting products and the length of the procedures varied greatly [11–13]. We hypothesized that the varied results of colony PCR for molecular detection were due to variations in the efficiency of disrupting the fungal cell wall and that colony PCR can still be applied for the detection of fungi, provided the number of DNA copies is sufficient, following improvements in the efficiency of cell wall-breaking methods.

The internal transcriber spacer (ITS) region is highly homologous among species of the genus* Trichosporon*, leading to the poor performance of molecular identification of* T. asahii* and difficulty in distinguishing it from closely related species. Thus, a region that has greater divergence among* Trichosporon* species is required for successful identification: namely, the intergenic spacer (IGS) shows greater genetic diversity than ITS in* Trichosporon*. Therefore, IGS, especially IGS1, has become the current gold-standard for accurate* Trichosporon* spp. identification [14].

Given the above background information and due to the lack of a specific, highly sensitive, rapid, and simple* T. asahii* identification method, in this study, we aimed to develop a colony PCR strategy that would directly identify fungal elements representing* T. asahii *in clinical samples. This method may help in the early detection and rapid diagnosis of* T. asahii* infections and facilitate appropriate treatment for patients.

## 2. Materials and Methods

### 2.1. Strains and Culture

We used 15* Trichosporon asahii *strains and 15 other* Trichosporon* strains, 28 non-*Trichosporon* fungal strains, and 9 bacterial strains (Table 1), to evaluate the sensitivity, positive rate, and primer specificity of the colony PCR strategy. Before the experiment, all strains were inoculated in potato dextrose agar or Luria–Bertani medium at 30–37°C for 48–72 h.

### 2.2. Suspension Preparation

To acquire a decimal concentration gradient of suspensions (10^7^, 10^6^, 10^5^, 10^4^, 10^3^, 10^2^, and 10^1^ CFU/mL), the suspension of 3.0 × 10^7^ CFU/mL was diluted 10-fold continuously. To confirm the concentration of spores, 20 *μ*L of the suspension with a concentration of 10^3^ CFU/mL was subcultured onto potato dextrose agar. The mean colony count on each plate was 45–75 CFU after 48 h of incubation.

### 2.3. DNA Extraction Methods

For the direct colony method, 1 *μ*L of suspension was used directly as the template. For the glass bead method, beads (400–600 *μ*m; Sigma-Aldrich, St. Louis, MO, USA) were added to 400 *μ*L of suspension, and the mixture was vortexed using a FastPrep®-24 Instrument (MP Biomedicals, USA) at a speed of 4 m/s for 20 s. After resting shortly, 1 *μ*L of supernatant was used as the template. For the freeze-thaw method, 400 *μ*L of the suspension was boiled at 100°C for 5 min, frozen at −80°C for 15 min, and then boiled at 100°C for 10 min. One microliter of the supernatant was used as the template. For the enzymolysis method, Snailase (Sigma-Aldrich) was added to 400 *μ*L of the suspension. The mixture was incubated at 37°C for 1 h, and 1 *μ*L of suspension was used as the template. For the traditional DNA extraction method, DNA was extracted from* T. asahii* per the CBS protocol “Extraction of ribosomal DNA according to Möller (modified).”

### 2.4. Primers and Design

In order to evaluate the efficiency of the DNA extraction methods, we chose the panfungal primers 26SF and 5SR to amplify partial 26S rDNA, IGS1, and partial 5S rDNA. Four species-specific primer pairs derived from the IGS1 region of ribosomal DNA (rDNA) were designed for* T. asahii* identification using Oligo software (v. 7.60). Target sequences were retrieved from the NCBI website (https://blast.ncbi.nlm.nih.gov/Blast.cgi). All primers were then synthesized by Sangon Biotech (Shanghai, China). The sequence of each primer is shown in Table 3.

### 2.5. PCR Conditions

PCR was performed in a total reaction volume of 25 *μ*L, containing 3 *μ*L of 10× buffer, 0.2 mM dNTPs, 0.6 mM MgCl_2_, 0.4 *μ*M of each primer, 1 U Taq DNA polymerase, and 1 *μ*L of DNA template. Thermal cycling consisted of 5 min at 94°C for initial denaturation, followed by 30 cycles of denaturation at 94°C for 45 s, annealing at 53°C for 45 s, and extension at 72°C for 75 s, with a final extension step at 72°C for 7 min. The PCR products were examined by electrophoresis on 1.5% w/v agarose gels.

### 2.6. Sensitivity Detection

To determine the sensitivity of each method, the serial dilutions of* T. asahii *CBS2479 were subjected to the different DNA extraction methods.

### 2.7. Positive Rate of Trace Detection

To evaluate the positive rate of trace detection of each method, suspensions of 15* T. asahii *strains, at a concentration of 10^3^ CFU/mL, were used.

### 2.8. Direct Detection of* T. asahii* from Clinical Samples Mimicking Infection

Clinical samples including whole blood, bronchoalveolar lavage fluid (BALF), and urine were collected from patients at our hospital. Suspensions of* T. asahii* CBS2479 were mixed with the clinical samples to a concentration of 10^7^ CFU/mL, and the mixtures were diluted 10-fold using the respective samples. Whole blood samples were lysed with Red Blood Cell (RBC) Lysis Buffer (Thermo Fisher Scientific, Waltham, MA, USA). All prepared samples were pretreated with glass beads and then directly used as templates for the PCR reaction.

### 2.9. Controls

A number of precautions were undertaken to control the occurrence of false-positive results. No-templates were processed together with the specimens tested. All experiments were performed in triplicate.

## 3. Results

### 3.1. Sensitivity of Colony PCR

**Figure 1:**
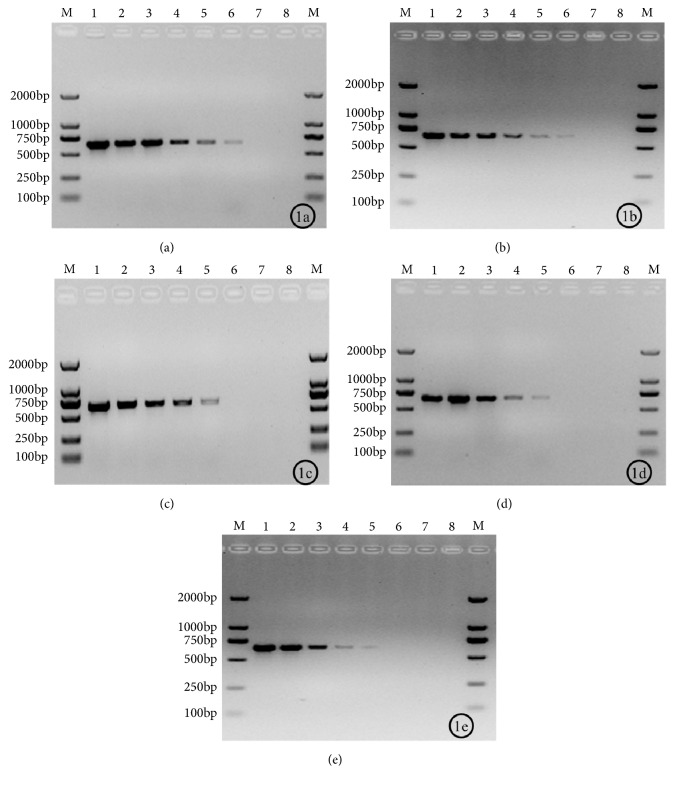
The sensitivity of colony PCR detection based on different methods. M: DNA Ladder; 1-7: Suspension of the type strain of* T. asahii *CBS2479, Concentration followed by 10^7^, 10^6^, 10^5^, 10^4^, 10^3^, 10^2^, 10^1^ CFU/mL; 8: Negative control; 1(a): glass beads; 1(b): freeze-thawing; 1(c): direct colony; 1(d): enzymolysis; 1(e): traditional DNA extraction.

The analytical sensitivity was determined by 10-fold serial dilutions of the* T. asahii* suspension. The minimal detection concentration of the glass bead and freeze-thaw methods was approximately 10^2^ CFU/mL (Figures 1(a), lane 6, and 1(b), lane 6). The direct colony and enzymolysis methods presented the sensitivity of 10^3^ CFU/mL (Figures 1(c), lane 5, and 1(d), lane 5). The minimal detection concentration of the traditional DNA extraction method was also 10^3^ CFU/ mL (Figure 1(e), lane 5). Similar results were obtained when the assay was repeated, demonstrating the reproducibility of the test.

### 3.2. Positive Rate of Trace Detection of Colony PCR

**Figure 2:**
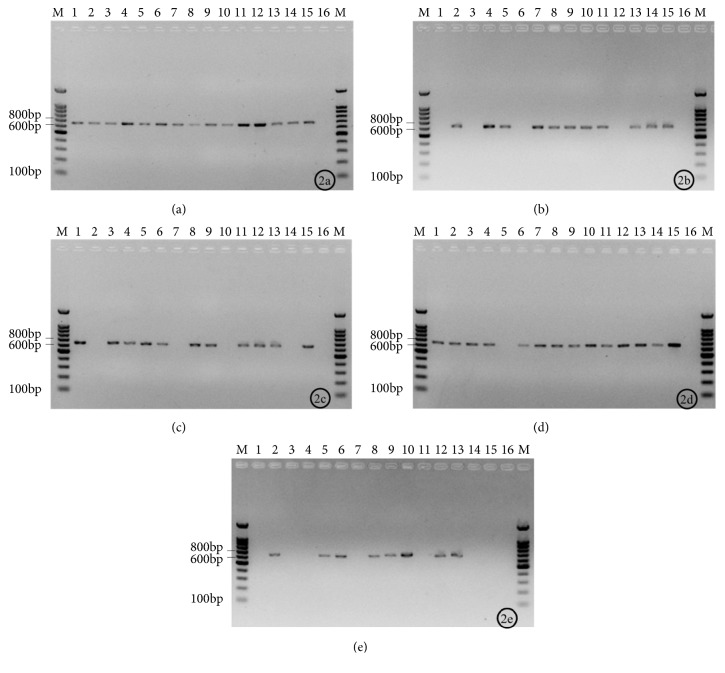
The positive rate of trace detection of colony PCR trace detection based on different methods for 15 isolates of* Trichosporon asahii*. M: DNA Ladder; 1: BZP7001; 2: BZP7002; 3: BZP7003; 4: BZP7004; 5: BZP7005; 6: BZP9001; 7: BZP9002; 8: CBS2479; 9: BZP4848; 10: BZP6108; 11: BZP6198; 12: BZP6674; 13: CBS7137; 14: CBS8520; 15: CBS8904; 16: Negative control; 2(a): glass beads; 2(b): freeze-thawing; 2(c): direct colony; 2(d): enzymolysis; 2(e): traditional DNA extraction.

In light of the above results, we chose the concentration of 10^3^ CFU/mL to evaluate the positive rate of trace detection of colony PCR. Fifteen strains of* T. asahii *yielded positive results by glass beads method (Figure 2(a)), presenting a positive rate of 100%. Eleven of the 15 strains yielded positive results by the freeze-thaw method (Figure 2(b)) and direct colony method (Figure 2(c)); i.e., the positive rate was 73.3%. The positive rate of the enzymolysis method was 93.3%, with 14 out of 15 strains being positive (Figure 2(d)). Only 8 of the 15 strains were shown positive by electrophoresis when the traditional DNA extraction method was used, i.e., positive rate was 53.3% (Figure 2(e)).

### 3.3. Procedure Duration

The duration of colony PCR was evaluated: it was significantly lower in the direct colony and glass bead groups, with 151 ± 0.3 min and 154.8 ± 0.6 min, respectively, compared to the freeze-thaw, enzymolysis, and traditional DNA extraction groups, which took 182.7 ± 1.5 min, 210.6 ± 0.4 min, and 445.4 ± 7.9 min, respectively (Table 2).

### 3.4. Specificity Test

Reference strains of species closely related to* T. asahii*, other fungal strains, and bacterial strains or human DNA extracted from healthy subjects or controls lacking genomic DNA yielded negative (no amplification) results when using the primers TA4F and TA4R. The other primers did not meet the specificity requirements (Table 1). Consequently, TA4F and TA4R, which showed perfect specificity, were selected for subsequent optimization of colony PCR.

### 3.5. Analysis of Infectious Samples

Colony PCR was performed on infectious samples. All of the samples tested positive, whereas the control group tested negative (Table 4). The colony PCR exhibited high sensitivity to whole blood, BALF, and urine samples mimicking infection that reach up to 10^2^ CFU/mL.

## 4. Discussion

*T. asahii* is the most commonly isolated species from patients with invasive trichosporonosis [9], and establishing a fast and effective molecular detection method would be helpful in the early clinical diagnosis of this infection.* T. asahii* has become the predominant species involved in systemic mycosis. The emergence of less common, but medically important opportunistic fungal pathogens has contributed to an increase in the rate of morbidity and mortality [15]. Due to the low immune status, multiple pathogenic organisms may coexist in the same patient. To eliminate the interference of other pathogenic organisms, high specificity of the identification method is particularly important. Colony PCR is widely used in various fields of microbiological science, including* Escherichia coli*,* Bacillus subtilis*,* Bacillus coagulans*,* Pichia pastoris*,* Candida*, and* Aspergillus* detection [11, 16, 17], and has the potential to be used as a simple detection assay on account of its simplicity, robustness, and low cost. Unfortunately, the application of colony PCR for the detection of* T. asahii *has not yet been researched due to the difficulty in cell-wall destruction during sample treatment. In this study, we set up and evaluated the performance of a colony PCR assay for the detection of* T. asahii* in culture and mimicking clinical samples.

Due to the cell-wall differences among the species, the quality of amplicons obtained by colony PCR differs in different pathogen studies. High quality and purity of extracted DNA is the foundation of reliable colony PCR. Therefore, the efficiency of cell wall break-down methods needs to be improved. The previous applications of colony PCR were based on pure colony culture, requiring an additional time of 24-48 hours or more, which greatly undermined the rapidity of the colony PCR technology [11, 13]. The above two problems have created an insurmountable gap between the colony PCR technology and the rapid clinical detection of pathogenic fungi. Only by bridging this gap can the advantages of this technology be truly applied in the clinical setting. To this end, our study has three highlight points: (1) analysis of the rapid and convenient release method of fungal DNA, and preliminary establishment of a colony PCR detection system with high sensitivity and simple operation methods; (2) development of the* T. asahii*-specific colony PCR detection method through design, screening, and specificity determination of species-specific primers, so that this method can be used to detect not only* T. asahii* but also other pathogenic fungi by simply changing the genus- or species-specific primers; (3) evaluation of the applicability of the* T. asahii*-specific colony PCR detection method to clinical samples.

In this study, we compared the efficiency of various methods for preliminary DNA extraction. The results showed that the sensitivity and positive rate of the glass beads method were 3 × 10^2^ CFU/mL and 100%, respectively, with a markedly low time consumption of 154.8 ± 0.6 min. One microliter of the suspension with a concentration of 3 × 10^2^ CFU/mL contained approximately 3 × 10^−1^ spores, which were detectable by PCR considering that each spore contains multiple copies of rDNA. In fact, according to a study, there are about 100–150 copies of rDNA genes of the yeast cell located in one chromosome of* Saccharomyces cerevisiae *[18, 19]. Therefore, 1 *μ*L of the suspension contained estimated 30–45 copies. On the other hand, the glass beads method has a high cell wall-broken efficiency, and the multiple copies of rDNA and high wall-broken efficiency yielded an adequate amount of DNA for amplification. Meanwhile, false-positive results can be avoided by aseptic conditions and by minimizing the length of DNA extraction to 20 s. These advantages are not offered by the other three methods in this study. The glass beads method can overcome the disadvantages of the previous colony PCR method in practical applications. Therefore, we decided to use the glass beads method as the colony PCR pretreatment strategy and initially established a highly sensitive, convenient, and easily operable colony PCR system.

ITS has been used to molecularly identify the microorganism, but the IGS1 region is considered more suitable for accurate* Trichosporon* species identification due to higher discriminatory power [20–22]. To accurately identify* T. asahii* strains to the species level, we designed 4 pairs of species-specific primers based on the IGS1 region of rDNA and successfully obtained the primers TA4F and TA4R that had satisfactory specificity. The primers TA4F and TA4R showed high specificity for IGS1 of* T. asahii* and did not amplify DNA of other common pathogenic fungi and bacteria. This method can detect various* T. asahii* strains using only colony PCR within 3 h, without fungal culture, DNA extraction, and sequencing for species identification. So far, we successfully established a* T. asahii*-specific detection method based on colony PCR, which is of great significance for the current situation regarding clinical* T. asahii* infection and the urgent requirement for a novel detection method.

PCR is often used to detect DNA extracted from pure culture colonies derived from infected samples, but seldom used to directly detect pathogens in clinical samples. In order to determine whether the complex components in clinical infection samples would interfere with the amplification efficiency of the PCR reaction and further observe the detection limit of colony PCR for clinical samples infected with* T. asahii*, we chose to use simulated clinical samples in this study. Considering that* T. asahii* seems to be common species isolated from the blood, BALF, and urine of hospitalized patients [9, 20, 23, 24], we used the species-specific colony PCR to investigate whole blood, BALF, and urine samples mimicking infection. The assay yielded positive results in whole blood, BALF, and urine mimicking human infection, with a high sensitivity of 10^2^ CFU/mL. These factors reinforce the need for methods that provide rapid and accurate identification of* T. *asahii from easily obtainable clinical samples, and the trace detection sensitivity of species-specific colony PCR fully meets these requirements.

Previous studies on the application of nested PCR in the detection of* T. asahii* in clinical samples reported some shortcomings, such as the need to sequence the amplification products to identify the pathomycete [25], or the selection of the ITS region with small interspecies differences as the amplification region [26]. Although several studies have reported the successful detection of* T. asahii* in different clinical samples by real-time quantitative PCR, the currently recognized IGS1 region was not selected for primer designing [27, 28]. This could lead to interspecies misinterpretation, if* Trichosporon* species are identified through the non-IGS1 region. There have also been reports on the design of species-specific primers for real-time quantitative PCR based on the IGS1 region for the detection of* T. asahii* in whole blood samples. However, the procedure required extraction of DNA from blood samples for amplification, which increased the total time of detection [29]. Our previous study on the detection of* T. asahii* using LAMP technology also showed requirement for DNA extraction after obtaining pure culture, and the cost of this technology was much higher than that of PCR, both of which hindered the application of this technology in clinical practice [8]. The* T. asahii*-specific colony PCR method for direct detection of the infection in clinical samples, established in this study, can overcome the drawbacks of the nucleic acid amplification technology mentioned above, so as to meet the need for rapid detection of pathogens in clinical practice and facilitate early treatment strategies.

The* T. asahii* detection method established in this study proved to be efficient and easily operable. This method does not require the use of expensive equipment or special kits or reagents. It can be widely carried out in developing countries or low-level medical institutions. At the same time, the technology has broad expansibility: the specific detection of other pathomycete can be efficiently performed by simply replacing the genus- or species-specific primers. In order to further shorten the detection time, we plan to demonstrate the feasibility of real-time quantitative colony PCR in our next study.

## 5. Conclusions

In conclusion, our study found that species-specific colony PCR, preceded by glass bead DNA extraction, could be used for the identification of* T. asahii*. In comparison with conventional methods, it produces results in a shorter time (within 3 h), requires less effort and resources, and is less expensive, rendering it suitable for diagnostic application. This methods applicability will enable medical institutions with poorer diagnostic capabilities to perform accurate diagnosis of* T. asahii* infection. At the same time, it can be extended to detect more pathogenic fungi and offer research scope to further shorten the detection time.

## Figures and Tables

**Table 1:** Strains used in this study and the specificity evaluation of species-specific primers for detecting *T. asahii.*

Organism	Strain	*Specificity detection*			
		TA1	TA2	TA3	TA4
*Trichosporon asahii*	BZP7001	+	+	+	+
*Trichosporon asahii*	BZP7002	+	+	+	+
*Trichosporon asahii*	BZP7003	+	+	+	+
*Trichosporon asahii*	BZP7004	+	+	+	+
*Trichosporon asahii*	BZP7005	+	+	+	+
*Trichosporon asahii*	BZP9001	+	+	+	+
*Trichosporon asahii*	BZP9002	+	+	+	+
*Trichosporon asahii*	CBS2479	+	+	+	+
*Trichosporon asahii*	BZP4848	+	+	+	+
*Trichosporon asahii*	BZP6108	+	+	+	+
*Trichosporon asahii*	BZP6198	+	+	+	+
*Trichosporon asahii*	BZP6674	+	+	+	+
*Trichosporon asahii*	CBS7137	+	+	+	+
*Trichosporon asahii*	CBS8520	+	+	+	+
*Trichosporon asahii*	CBS8904	+	+	+	+
*Trichosporon debeurumanianum *	CBS1896	−	−	−	−
*Trichosporon dermatis *	CBS2043	−	−	−	−
*Trichosporon cutaneum *	CBS2466	−	−	−	−
*Trichosporon asteroides *	CBS2481	−	+	+	−
*Trichosporon coremiiforme *	CBS2482	−	+	+	−
*Trichosporon faecale *	CBS4828	−	+	+	−
*Trichosporon inkin *	CBS5585	−	−	−	−
*Trichosporon jirovecii *	CBS6864	−	−	−	−
*Trichosporon ovoides *	CBS7556	−	−	−	−
*Trichosporon mucoides *	CBS7625	−	−	−	−
*Trichosporon montevideense *	CBS8261	−	+	+	−
*Trichosporon domesticum *	CBS8280	−	−	−	−
*Trichosporon japonicum *	CBS8641	−	+	−	−
*Trichosporon lactis *	CBS9051	−	−	−	−
*Trichosporon dohaense *	CBS10761	−	−	−	−
*Candida dubliniensis*	CMCC(F)C8f	−	−	−	−
*Candida tropicalis*	CMCC(F)C2f	−	−	−	−
*Candida parapsilosis*	CMCC(F)C4f	−	−	−	−
*Candida glabrata*	CMCC(F)T10a	−	−	−	−
*Candida metapsilosis*	CMCC(F)C4k	−	−	−	−
*Candida albicans*	ATCC90028	−	−	−	−
*Cryptococcus neoformans*	CMCC(F)D2a	−	−	−	−
*Cryptococcus gattii*	CMCC(F)D2e	−	−	+	−
*Trichophyton rubrum*	CMCC(F)T1h	−	−	−	−
*Trichophyton violaceum*	BZP1501	−	−	−	−
*Trichophyton mentagrophytes*	CMCC(F)T5e	−	−	−	−
*Malassezia furfur*	CMCC(F)T17a	−	−	−	−
*Malassezia sympodialis*	CMCC(F)T14a	−	−	−	−
*Sporothrix schenkii*	CMCC(F)D1a	−	−	−	−
*Microsporum gypseum*	CMCC(F)M2b	−	−	−	−
*Microsporum canis*	CMCC(F)M3h	+	−	−	−
*Aspergillus versicolor*	CMCC(F)A5c	−	−	−	−
*Aspergillus fumigatus*	CMCC(F)A1g	+	−	−	−
*Aspergillus niger*	CMCC(F)A3a	−	−	−	−
*Aspergillus nidulans*	CMCC(F)A7c	−	−	−	−
*Fonsecaea pedrosoi*	CMCC(F)D6j	+	−	−	−
*Phialophora verrucosa*	CMCC(F)D8i	−	−	−	−
*Scedosporium apiospermum*	CMCC(F)D13f	−	−	−	−
*Penicillium marneffei*	CMCC(F)B33a	−	−	−	−
*Rhizopus oryzae*	CMCC(F)B81c	−	−	−	−
*Mucor circinelloides*	CMCC(F)B57a	−	−	−	−
*Fusarium oxysporum*	CMCC(F)B38c	−	−	−	−
*Absidia Corymbifera*	CMCC(F)B69f	−	−	−	−
*Staphylococcus aureus*	Clinical isolates	−	−	−	−
*Staphylococcus epidermidis*	Clinical isolates	−	−	−	−
*Streptococcus pneumoniae*	Clinical isolates	−	−	−	−
*Streptococcus pyogenes*	Clinical isolates	−	−	−	−
*Klebsiella pneumonia*	Clinical isolates	−	−	−	−
*Escherichia coli*	Clinical isolates	−	−	−	−
*Acinetobacter baumannii*	Clinical isolates	−	−	−	−
*Enterococcus faecium*	Clinical isolates	−	−	−	−
*Serratia odorifera*	Clinical isolates	−	−	−	−
Healthy human DNA		−	−	−	−
Negative control		−	−	−	−

ATCC: American type culture collection, Rockville, MD, USA; CBS: Centraalbureau voor Schimmelcultures, Baarn, The Netherlands; CMCC: China Medical Culture Collection, Nanjing, China; BZP: The Military Institute of Injury and Reparation, China.

**Table 2:** Sensitivities, positive rate of trace detection, and procedure duration of different colony PCR methods for detecting *T. asahii.*

Methods	Sensitivity	Positive rate of trace detection	Procedure duration			
			Pretreatment	PCR	Electrophoresis	Total
glass beads	10^2^CFU/mL	100% (15/15)	4.8±0.6min			154.8±0.6min
freeze-thawing	10^2^CFU/mL	73.33% (11/15)	32.7±1.5min			182.7±1.5min
direct colony	10^3^CFU/mL	73.33% (11/15)	1±0.3min	100min	50min	151±0.3min
enzymolysis	10^3^CFU/mL	93.33% (14/15)	60.6±0.4min			210.6±0.4min
traditional DNA extraction	10^3^CFU/mL	53.33% (8/15)	295.4±7.9min			445.4±7.9min

**Table 3:** Primers used in this study.

Primer Name	Sequence (5′-3′)
26SF	ATCCTTTGCAGACGACTTGA
5SR	AGCTTGACTTCGCAGATCGG
TA1F	GTGAATCAAGAACGAAGTATAAGGG
TA1R	AGACCTCAGCCTCTGACAGC
TA2F	CCTTTTGGACTCTCTATGATTGG
TA2R	CCATCCTTCCAACTTGTAGCTT
TA3F	TTGGACTCTCTATGATTGGCA
TA3R	TCTAGTCCTCAACCGCCCT
TA4F	GCGACCTCAGCATCTTAATCA
TA4R	CTCTGAGGCCTTGCTCCTGT

**Table 4:** Results of colony PCR in clinical samples mimicking infection.

Sample type	PCR results			
	Infectious samples		Noninfectious samples	
	No. of samples	No. positive (%)	No. of samples	No. positive (%)
Human, whole blood	10	10 (100)	10	0 (0)
Human, bronchoalveolar lavage	10	10 (100)	10	0 (0)
Human, urine	10	10 (100)	10	0 (0)

## Data Availability

No data were used to support this study.
